# 3D Nanoporous Anodic Alumina Structures for Sustained Drug Release

**DOI:** 10.3390/nano7080227

**Published:** 2017-08-21

**Authors:** Maria Porta-i-Batalla, Elisabet Xifré-Pérez, Chris Eckstein, Josep Ferré-Borrull, Lluis F. Marsal

**Affiliations:** Departament d’Enginyeria Electrònica, Elèctrica i Automàtica, ETSE, Universitat Rovira i Virgili, Avda. Països Catalans 26, 43007 Tarragona, Spain; maria.porta@urv.cat (M.P.-i.-B.); elisabet.xifre@urv.cat (E.X.-P.); chris.eckstein@urv.cat (C.E.); josep.ferre@urv.cat (J.F.-B.)

**Keywords:** drug delivery, nanoporous anodic alumina, complex pore geometry, release rate, nanotechnology

## Abstract

The use of nanoporous anodic alumina (NAA) for the development of drug delivery systems has gained much attention in recent years. The release of drugs loaded inside NAA pores is complex and depends on the morphology of the pores. In this study, NAA, with different three-dimensional (3D) pore structures (cylindrical pores with several pore diameters, multilayered nanofunnels, and multilayered inverted funnels) were fabricated, and their respective drug delivery rates were studied and modeled using doxorubicin as a model drug. The obtained results reveal optimal modeling of all 3D pore structures, differentiating two drug release stages. Thus, an initial short-term and a sustained long-term release were successfully modeled by the Higuchi and the Korsmeyer–Peppas equations, respectively. This study demonstrates the influence of pore geometries on drug release rates, and further presents a sustained long-term drug release that exceeds 60 days without an undesired initial burst.

## 1. Introduction

A new generation of local drug release platforms with sustained and complex release profiles for reduced therapeutic doses has emerged to overcome the disadvantages of conventional treatments—generally oral or intravenous administrations—with considerable adverse effects [1,2].

These local drug release platforms face important challenges to ensure efficient therapy: (i) efficient loading of drugs, (ii) sustained delivery, (iii) avoiding the ‘burst effect’ (high dose release within the first minutes), (iv) material stability (avoiding degradation), and (v) the possibility to chemically modify their surface for a selective release [3,4,5,6,7,8]. Many types of materials are currently used for the development of these new drug delivery platforms, such as polymers [9], hydrogels [10,11], iron oxide [12], graphene [13], porous silicon [7], and mesoporous silica [14]. However, most of these existing carrier materials rapidly degrade at physiological pH or/and show poor drug loading with drugs mainly attaching to external surfaces and leading to an intense initial “burst” release.

Porous materials have attracted great interest for the development of controlled drug delivery platforms because of their high effective surface area and tunable pore size [15]. The pore geometry is one of the main determining factors of the total drug load entering the pores and the release profile. Three-dimensional (3D) pore structures, with intricate pore geometries and increasing surface area are promising platform designs for sustained drug release. However, their fabrication can be expensive and complex.

Nanoporous anodic alumina (NAA), readily and cost-effectively fabricated by electrochemical anodization, permits obtaining elaborate and reproducible 3D pore geometries. The many physical and chemical properties of NAA make this material a versatile and interesting platform for controlled drug release. NAA has a highly ordered pore distribution, and its well-known electrochemical fabrication techniques allow for the precise control of pore diameter, interpore distance, pore length, and pore geometry [16,17,18]. NAA is highly stable at physiological pH, and has been successfully used in a wide array of medical and biological applications like orthopedic prosthetics, dental and coronary stents, cell culture scaffolds, immunoisolation devices, and biomolecular filtration [2,19,20,21].

Its high effective surface area makes NAA an ideal material for drug delivery applications providing pores as nanocontainers with regular and controlled structural features for loading active agents like drugs or molecules [22,23]. Moreover, the surface of NAA can be functionalized to be selective for specific molecules and covered with biodegradable, chemical, or pH responsive agents to trigger and regulate the release [24,25,26].

Although drug release from nanoporous coatings has already been studied, there is a lack of understanding of the release kinetics from NAA platforms with complex pore geometries and the dynamics governing them [27].

In this work the drug release kinetics for simple and complex NAA pore structures is investigated. Pores with straight walls and 3D pore structures with multilayered funnel and inverted funnel geometries are fabricated by electrochemical anodization, resulting in complex NAA platforms for drug delivery. Using the chemotherapeutic Doxorubicin, the drug release of these different pore geometries is studied, and the release mechanism is modeled by mathematical expressions.

## 2. Materials and Methods

### 2.1. Fabrication of NAA Structures

All NAA porous structures were prepared by electrochemical anodization of high purity (99.999%) aluminum plates (Goodfellow, Huntingdon, UK) in phosphoric acid electrolyte. The aluminum plates were initially degreased with acetone and ethanol to eliminate organic impurities and electropolished in a mixture of perchloric acid and ethanol 1:4 (*v*/*v*) at a constant voltage of 20 V for 6 min. To suppress breakdown effects and to enable uniform oxide film growth under hard anodization conditions (194 V in phosphoric acid at −5 °C), a protective layer was pre-anodized at a lower voltage (174 V in phosphoric acid) for 180 min [28]. Subsequently, the voltage was ramped up to 194 V at a constant rate of 0.05 V/s, and anodized for 20 h. After this first anodization step, the formed NAA layer was removed by wet chemical etching in a mixture of phosphoric acid (0.4 M), and chromic acid (0.2 M) at 70 °C for 4 h, resulting in a hexagonally-ordered pattern of the aluminum surface [29,30].

For all pore structures, the subsequent anodization steps were performed under hard anodization conditions, and the length of the pores was accurately controlled via the total charge.

**Figure 1:**
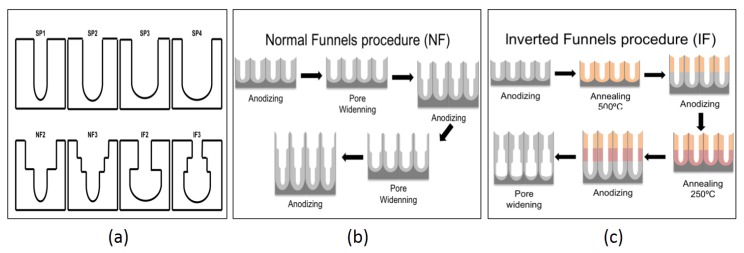
(**a**) Schematic illustration and labeling of the different pore geometries; (**b**) Schematics of the fabrication procedures for normal funnels; and (**c**) inverted funnels.

A single hard anodization step was performed to obtain straight pores (SP) with a uniform pore diameter from top to bottom (Figure 1a). The length of the pores of all SP is 30 µm. To widen the pores, wet chemical etching with aqueous solution of 5% H_3_PO_4_ was performed for 0 (SP1), 45 (SP2), 90 (SP3), and 120 min (SP4).

Normal Funnels (NF) were produced by a sequential combination of hard anodization and pore widening steps, and labeled according to the final number of layers. NF2 consist of a 15 µm thick top layer, widened in 5% H_3_PO_4_ for 90 min, and a 15 µm thick bottom layer. Similarly, NF3 consist of a 10 µm thick top layer widened for a total of 90 min (2 × 45 min), a middle layer of 10 µm widened for 45 min, and a bottom layer of 10 µm (Figure 1b).

The fabrication of Inverted Funnels (IF) required a thermal treatment at 250 °C and 500 °C to change the crystallographic phase of the alumina. The inverted funnels were labeled IF2 and IF3 according to their respective total number of layers (Figure 1c). IF2 consisted of a top layer of 15 µm, followed by a thermal treatment at 500 °C. A subsequent anodization step added a 15 µm thick bottom layer. Similarly, IF3 consisted of 3 layers, each with a thickness of 10 µm. After the anodization of the top and middle layer a thermal treatment of 500 °C and 250 °C was applied, respectively. In a final wet chemical etching step, the pores of IF2 and IF3 were widened for 2 h.

All NAA platforms were cut with a circular cutter to obtain samples with the same diameter (4 mm). All the experiments were conducted in triplicates.

### 2.2. Characterization of NAA Structures

All NAA structures were characterized by Environmental Scanning Electron Microscopy (ESEM, FEI Quanta 600, FEI Co., Hillsboro, OR, USA). The wet chemical etch rate during the pore widening steps was estimated for samples with and without a 500 °C thermal treatment. For the calibration of the pore widening process, ESEM images were taken in 15 min etching intervals, and the pore diameter was estimated using a standard image processing package (ImageJ, version 1.51p, public domain program developed at the RSB of the NIH, Bethesda, Maryland, MD, USA) (Figure S1).

### 2.3. Drug Loading and Release

Doxorubicin (DOX), a self-fluorescent chemotherapeutic agent, was selected as a model drug. Drug loading into NAA pores was performed through capillary action by immersing NAA into a DOX solution of 1 mg/mL. The suspension was stirred overnight in the dark with the NAA structures immersed. Subsequently, samples were washed with deionized water to remove any residual drug molecules on the surface of the sample and dried at an ambient temperature.

The release studies were performed in vitro using phosphate-buffered saline (PBS), which is commonly employed to simulate in vivo conditions for drug release. DOX release was estimated by directly measuring the photoluminescence of the release medium. This in situ measurement process is ideal to understand the release kinetics and the short-term release effect since it allows for the fast and frequent collection of release data. Samples were immersed in 0.5 mL of PBS which was renewed after every measurement. The fluorescence of the buffer solution was measured at regular time intervals at room temperature using a fluorescence spectrophotometer from Photon Technology International Inc. (Birmingham, NJ, USA), with an Xe lamp as the excitation light source, an excitation wavelength of 480 nm and an emission wavelength of 590 nm. The drug release was monitored by DOX photoluminescence over 65 days. The fluorescence intensities were converted to the corresponding concentrations using a calibration curve. All the drug release measurements were taken in triplicates for every pore structure and statistical analysis was performed.

## 3. Results and Discussion

**Figure 2:**
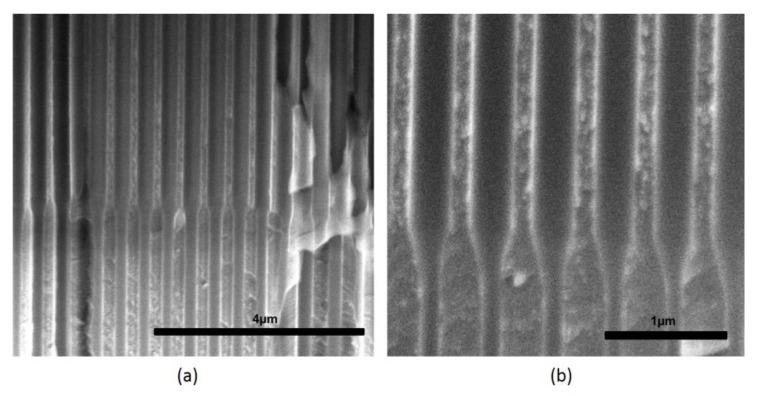
Cross-section Environmental Scanning Electron Microscopy (ESEM) images of a two layered normal funnel structure (NF2) illustrating (**a**) the uniform pore growth, and (**b**) the pore transition.

Normal Funnels (NF) with two and three layers of different pore diameters were successfully fabricated. ESEM cross-section images of NF show straight pore growth with no discontinuities (i.e., occluded pores) despite the interruption of the anodization process between layers (Figure 2). The transition between adjacent layers of different pore sizes is smooth and shows a conical shape. The pore diameter and the thickness of each NF layer was estimated from ESEM images and summarized in Table S1.

**Figure 3:**
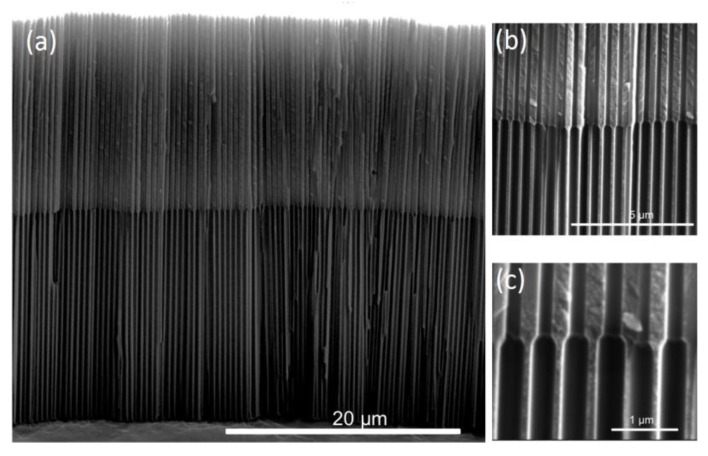
Cross section ESEM images of a two layered inverted funnel structure (IF2): (**a**) entire bilayer, (**b**) detail of the uniform pore growth, and (**c**) detail of the pore transition.

**Figure 4:**
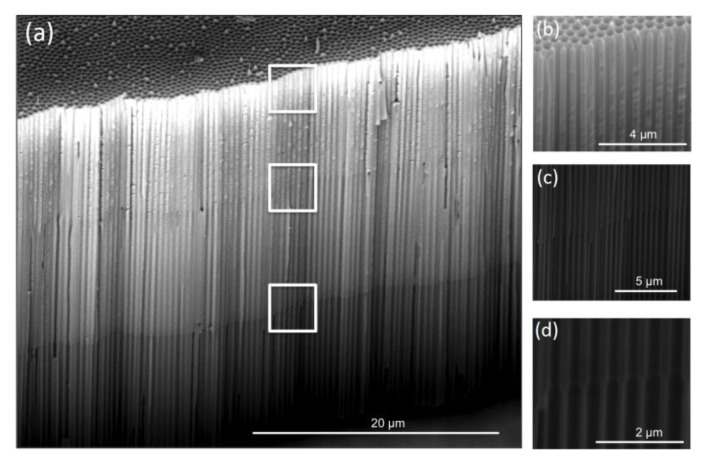
Cross section ESEM images of a three layered inverted funnel structure (IF3): (**a**) entire length of the inverted funnels, and (**b**–**d**) magnifications of the framed areas from top to bottom.

Inverted Funnels (IF) were also successfully fabricated, and their ESEM cross-section images are shown in Figure 3 (IF2) and Figure 4 (IF3). For both IF, parallel and perpendicular growth of the pores as well as conic and clear transitions between adjacent layers of different pore diameters can be observed. High magnification images show that these conic transitions between layers are more abrupt for IF than for NF. IF, NF, and SP were anodized to a total length of 30 µm for better comparability (Table S1).

The two distinct pore diameters of the IF2 top and bottom layers suggest that the crystallographic structure of the top layer was successfully modified by the temperature treatment (500 °C). The same clear transitions between layers are observed in IF3 samples, demonstrating that the intermediate annealing temperature of 250 °C results in an intermediate pore widening rate.

The effect of the temperature treatment on the pore widening process during IF fabrication was further assessed, and a calibration of the pore widening rates was determined. The pore diameters were estimated from ESEM images taken after consecutive 15 min pore widening steps for samples thermally treated at 500 °C, and for untreated samples. Figure S1 reveals that the thermally treated samples have a slower pore widening rate than untreated samples. The alumina matrix of untreated samples remained intact for up to 2 h of etching, but started to deteriorate after 2.5 h, and fully collapsed after 3 h due to over-etched pore walls. Consequently, to preserve full structural integrity of the thermally untreated samples, the pore widening was terminated after a maximum of 2 h. In contrast, the pore structure of thermally treated samples remains intact even after 3 h of etching.

Figure S2 shows the estimated pore diameter as a function of pore widening time, revealing higher etch rates for the untreated samples (2.5 nm/min) than for the thermally treated samples (1.2 nm/min). These are brought about by the thermal annealing, which increases the crystallinity, and consequently, the stability of the alumina, and also further promotes anion diffusion [31]. We further notice that the pore diameter linearly increases with the pore widening time until an inflection point, where the pore widening rate decreases considerably. This inflection point corresponds to an interface separating an outer region with concentrated anionic species from an inner region composed of pure alumina. The anion contaminated region is easily removed by the pore widening, whereas the region of pure alumina is more resistant to pore widening [32,33]. The inflection point is observed after 90 min and 150 min of pore widening for non-treated samples and thermally treated samples, respectively.

**Figure 5:**
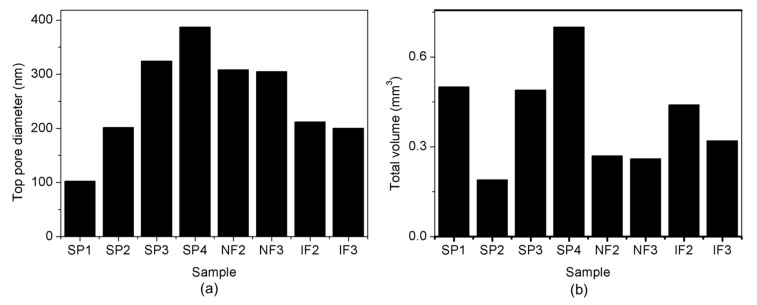
(**a**) Top pore diameter, and (**b**) total volume of the different samples.

Figure 5a shows the pore diameter of the top layers of all the fabricated NAA structures. Increasing pore diameters of samples SP1–SP4 are directly related to the increasing widening time intervals (Table S1). NF2 and NF3 show top pore diameters similar to SP3 (around 300 nm), and IF2 and IF3 top diameters are similar to that of SP4 (around 200 nm). These similarities allow for studying the influence of the pore geometry on the release kinetics. Figure 5b shows the total volume of all of the samples. The pore volumes of SP1–SP4 are clearly related to the pore diameters, whereas the pore volumes of the layered samples NF and IF depend on the complex geometries of the pores.

**Figure 6:**
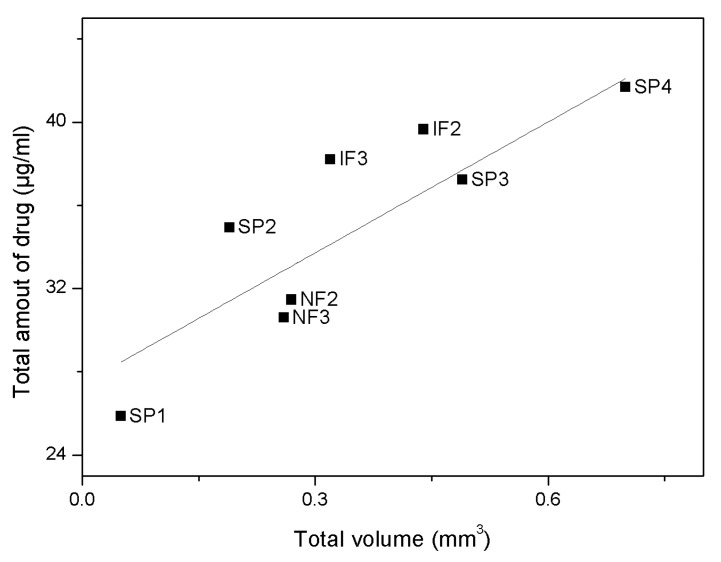
Total drug load within the pores versus the respective total volume for various nanoporous anodic alumina (NAA) structures. Linear regression for straight pores (SP) samples (dotted line).

The relationship between the total amount of drug load and volume is shown in Figure 6. The total drug load of straight pores is linearly proportional to the pore volume, as indicated by the linear regression for SP1–SP4 (dotted line). Similarly, the total drug load of NF follows the trend of SP samples, though, with an increasing diversion from this trend with more funnel layers. Interestingly, the IF samples seem to hold a higher drug load per pore volume when compared to SP, indicating an influence of the pore geometry on the total drug load. This can be explained by the contour of the IF pores which encompasses small pore diameters of the top layer, wider pore diameters of deeper layers and a sharp elbow-like transitions between the adjacent layers (Figure 3c). This intricate geometry retains a higher total drug load within the pores, making IF structures more efficient for drug loading than SP structures.

**Figure 7:**
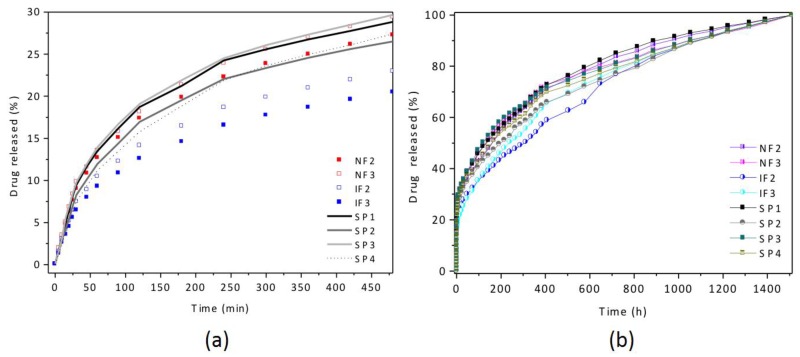
Cumulative drug release of different pore structures: (**a**) short-term and (**b**) long-term.

The influence of the pore geometry on short and long-term drug release has been studied for all fabricated NAA structures. Figure 7 and Figure S3 show the drug release response for the first 8 h, and for the complete release time of 1512 h (63 days). All of the NAA structures presented in this work can be considered as sustained drug delivery platforms due to very long drug release times. Interestingly, these NAA structures do not present a high initial drug release burst in the first minutes, in contrast to most drug delivery platforms in the literature [34,35,36]. The absence of an initial release burst indicates that the drug delivery from these NAA platforms is more constant in time, and prevents an undesired high initial dosage. Both the sustained delivery, and the absence of initial burst are relevant and differentiating properties and address two of the main challenges of localized drug delivery.

The drug release profile of all the pore geometries can be described by distinguishing two phases: (i) a short-term release with a higher release rate within the first 8 h, and (ii) a slow and sustained release where almost the entire drug load is delivered from the NAA after 63 days.

Most of the NAA structures presented here release only around 25–30% of the total drug load during the short-term release, which is very low compared to the 80% and above of most conventional structures in the literature [37,38]. Generally, the initial release is attributed to the fast diffusion of drug molecules residing on the NAA surface, rather than the diffusion of molecules attached to the walls within the pores. Here, this low short-term release indicates that most of the drug was loaded inside the pores during the incubation period.

The pore geometries were found to influence the short-term release rates. The 200 nm pore diameter of the top layer of IF2 and IF3 is similar to SP2, however, their release rates are considerably lower than SP2. The pore opening of IF acts like a bottleneck for the infiltrating medium and the eluting drug, hindering the circulation of the medium inside the pore and slowing down the diffusion of the drug out of the alumina. This effect augments with increasing IF layers: the release rate of IF3 is lower than that of IF2.

On the contrary, the NF and SP3, with very close top pore diameters of around 310 nm, present similar release profiles, the geometry of NF does not hinder the circulation of the medium inside of the pores.

The experimental data for short- and long-term release were modeled to measure the kinetics and establish the mechanism of DOX release. For short-term release, the experimental data was modeled using a variation of the Higuchi equation [9,39,40,41]:

(1) $$M_{t}=M_{0}+K\sqrt{t}$$

where *M_t_* is the cumulative release at time *t*, *M*_0_ is the intercept value at *t* = 0 and, *K* is the release constant that indicates the release velocity.

**Figure 8:**
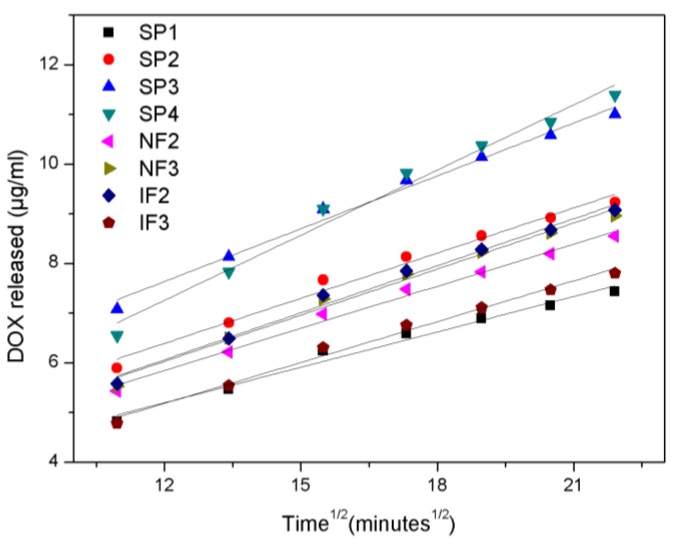
Short-term release data (symbols) and fitting (lines) using the Higuchi equation.

**Figure 9:**
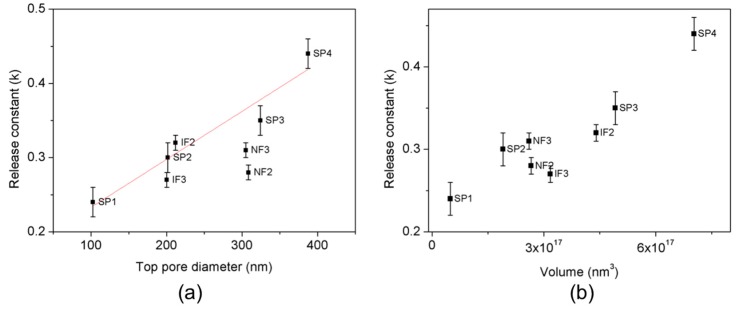
Release constant *K* during the short-term release versus (**a**) top pore diameter and, (**b**) volume. The red line corresponds to the linear fitting.

The fitting of the experimental data for all pore geometries with Equation (1) is presented in Figure 8, where the cumulative DOX release is plotted against the square root of time during the short-term release. The fitting is in very good agreement with the experimental data for all pore geometries, demonstrating that the drug kinetics can be approximated by the square root of time. Table 1 shows the fitting parameters for each pore structure and Figure 9 depicts their release constant *K* depending on the top pore diameter and volume.

During the short-term release, the release constant *K* for straight pore structures (SP1–SP4) is linearly proportional to the pore diameter, following the equation:

*K* = 0.168 + 6.46 × 10^−4^·*D_p_* (2)

where *K* is the release constant in (µg/mL)/min^1/2^ and *D_p_* is the pore diameter in nanometers.

IF are the structures with the highest load efficiency, as they retain a higher quantity of drug inside of the pores than SP and NF, with the same volume or top pore diameter. During short-term release, IF structures show a lower release constant *K* than SP structures with the same top pore diameter. However, if the specific application requires a higher release rate, SP structures are favorable. Even though SP and NF present similar release rates, the fabrication of SP structures is not as complex as the fabrication of NF.

For the long-term release the experimental data was modeled with the Korsmeyer–Peppas equation [6,38,42]:

(3) $$M_{t}=M_{t0}{\left(\frac{t}{t_{0}}\right)}^{n}$$

where *M_t_* is the quantity of drug released at time *t*, *M_t_*_0_ is the amount of drug released at the reference time *t*_0_ (day 1), *t* is time in days, and *n* is the release parameter related to the release rate.

**Figure 10:**
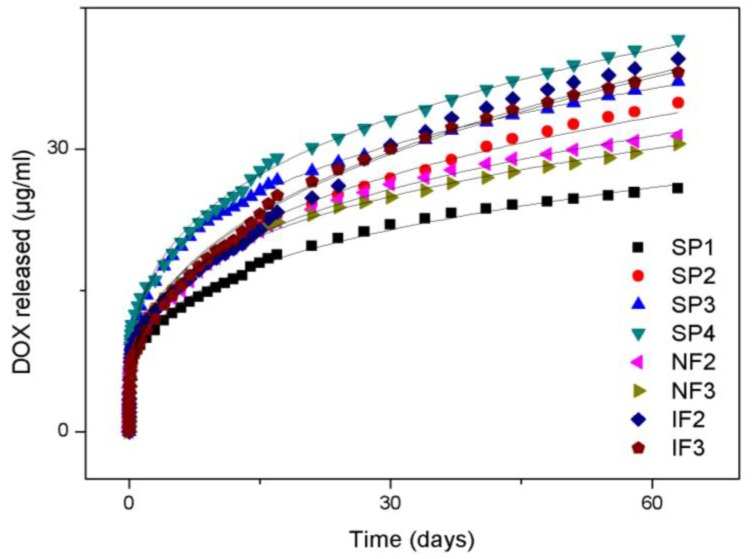
Long-term release experimental data (symbols) and fitting using the Korsmeyer–Peppas equation (lines).

Table 2 shows the values of these parameters fitted for the release of all the pore structures. The release rate was calculated with the first derivative of Equation (3) [3,22]. Figure 10 shows a good agreement between the experimental data and the fitting modeled with Equation (3).

**Figure 11:**
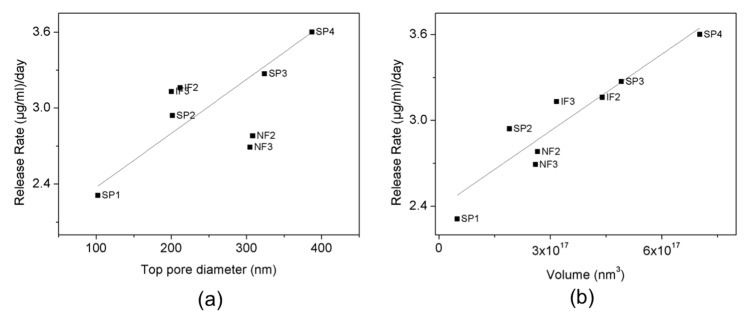
Release rate versus (**a**) top pore diameter, and (**b**) volume, and linear fittings (black straight line).

For the long-term release, a linear relation between the release rate and the pore diameter is observed for SP (Figure 11):

*Release rate* = 1.95 + 0.004*D_p_* (4)

where *D_p_* is the top pore diameter.

NF and SP3 have a similar top pore diameter and release rates. IF have slightly higher release rates than SP2, which is explained by the quantity of drug remaining inside the pores. During the short-term release, NF and SP delivered a higher part of their load than IF as their release rates were higher. Due to this, and the fact that drug loads were completely released from all the structures after 63 days, IF released greater loads during days 8–63 than the other structures, and therefore their release rate is slightly higher.

## 4. Conclusions

In this study, NAA platforms with a variety of 3D pore morphologies were fabricated, loaded with DOX, and the release mechanism was modeled by mathematical expressions. Besides, the influence of the pore geometry on the drug release kinetics was assessed.

The release profiles for the studied pore geometries revealed two interesting and promising properties: (i) very long drug release times determining the presented NAA as sustained drug delivery platforms, and (ii) a constant drug delivery free of an initial release burst to prevent undesired high initial drug delivery dosages. These findings are advancements to two of the main challenges of current platforms for advanced drug delivery systems.

The obtained results reveal that the pore geometry influences the total drug load within the pores. IF retain a higher quantity of drug inside the pores than SP and NF with the same volume or top pore diameter. The pore geometry also influences the release kinetics. During the short-term release, IF showed lower release rates than SP with the same top pore diameter.

Moreover, the dynamics of the release of all the pore structures were successfully modeled, and two different release regimes were differentiated: a short-term and a long-term release. The short-term release (first 8 h) was modeled by the Higuchi model, whereas for the long-term release the Korsmeyer–Peppas equations were used.

## Figures and Tables

**Table 1:** Higuchi equation fitting parameters.

Sample Name	Release Constant (*K*)	Intercept (*M*_0_)
SP1	0.24 ± 0.02	2.34 ± 0.26
SP2	0.30 ± 0.02	2.75 ± 0.29
SP3	0.35 ± 0.02	3.38 ± 0.29
SP4	0.44 ± 0.02	2.01 ± 0.43
NF2	0.28 ± 0.01	2.45 ± 0.21
NF3	0.31 ± 0.01	2.31 ± 0.25
IF2	0.32 ± 0.01	2.28 ± 0.25
IF3	0.27 ± 0.01	1.89 ± 0.20

**Table 2:** Korsmeyer–Peppas equation fitting parameters.

Sample Name	*M_t_*_0_ (μg/mL)	*n*	Release Rate
SP1	8.43 ± 0.14	0.27 ± 0.01	2.31
SP2	9.93 ± 0.21	0.30 ± 0.001	2.94
SP3	12.49 ± 0.17	0.26 ± 0.00	3.27
SP4	12.55 ± 0.22	0.28 ± 0.01	3.60
NF2	9.70 ± 0.16	0.29 ± 0.00	2.78
NF3	10.14 ± 0.15	0.27 ± 0.00	2.69
IF2	9.00 ± 0.33	0.35 ± 0.01	3.16
IF3	8.91 ± 0.18	0.35 ± 0.01	3.13

## Supplementary Materials

The following are available online at http://www.mdpi.com/2079-4991/7/8/227/s1. Figure S1: Pore widening progress for samples with and without temperature treatment; Figure S2: Pore widening calibration for samples with and without thermal treatment; Figure S3: Cumulative drug release of different pore structures; Table S1: Average dimensions of the pore structures.
