# Supplementary material for: 3D Nanoporous Anodic Alumina Structures for Sustained Drug Release
Source: Nanomaterials (Basel). 2017 Aug 21;7(8):227. doi: 10.3390/nano7080227 (PMC5575709; doi:10.3390/nano7080227)
Supplement: Supplementary file 1 [file nanomaterials-07-00227-s001.pdf]

Supplementary Information

3D Nanoporous Anodic Alumina Structures for Sustained Drug Release

MARIA PORTA-I-BATALLA, ELISABET XIFRE-PEREZ, CHRIS ECKSTEIN,

JOSEP FERRÉ-BORRULL, LLUIS F. MARSAL

Nano-electronic and Photonic Systems (NePhoS)

Departament d'Enginyeria Electrònica, Elèctrica i Automàtica

Universitat Rovira i Virgili.

Avda. Països Catalans 26

43007 Tarragona, Spain.

Figure S1. Pore widening progress for samples with and without temperature treatment.

ESEM images of NAA taken every 15 min of pore widening.

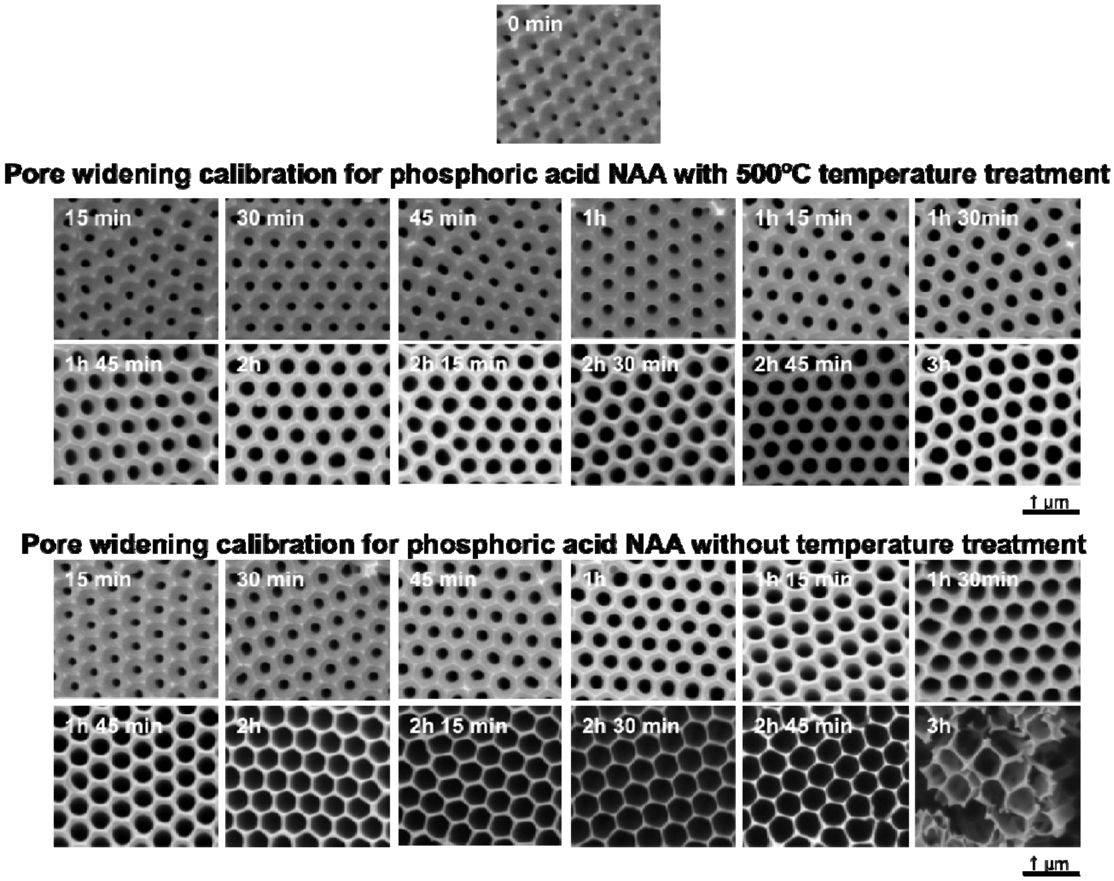

Figure S2. Pore widening calibration for samples thermally treated at 500 °C (red circles) and samples without temperature treatment (black squares).

Pore widening rate is calculated for the region where the relationship between pore diameter and pore widening time is linear (0 min to inflection point time):

- No thermal treatment: from 0 to 90 min.

- 500 °C thermal treatment: from 0 to 150 min.

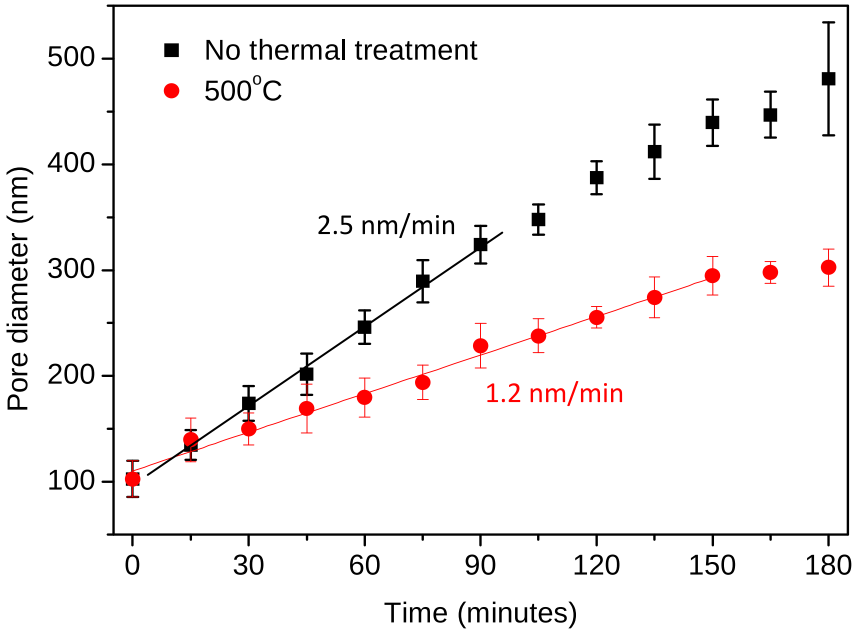

Figure S3. Cumulative drug release of different pore structures.

(A) Release from straight pores (SP), and (B) release from Normal Funnels (NF2, NF3) and from Inverted Funnels (IF2, IF3).

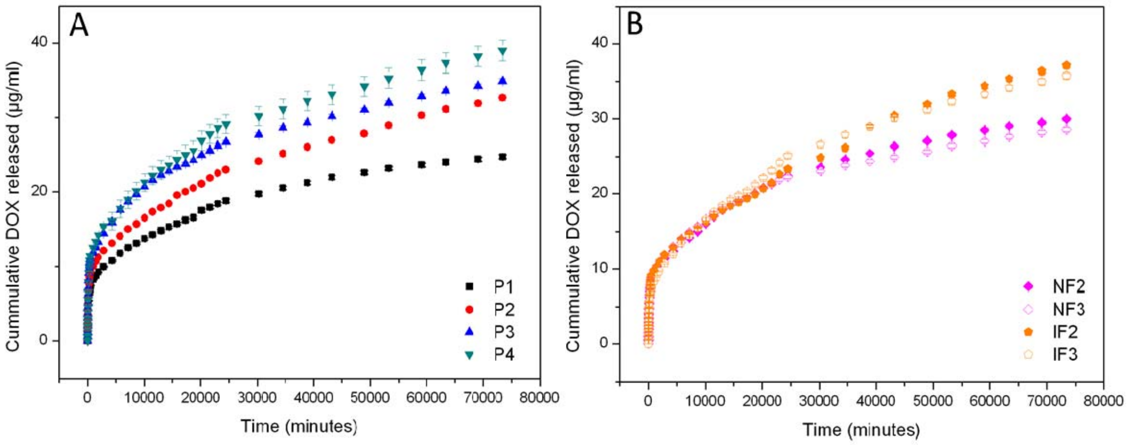

Table S1. Average dimensions of the pore structures.

Sample	Widening time (minutes)	Top pore diameter (nm)	Middle pore diameter (nm)	Bottom pore diameter (nm)	Top height (µm)	Middle height (µm)	Bottom height (µm)	Total volume (nm^3)
**SP1**	0	102,5	-	-	30	-	-	4,9 E+16
**SP2**	45	201,6	-	-	30	-	-	1,9 E+17
**SP3**	90	324,2	-	-	30	-	-	4,9 E+17
**SP4**	120	387,2	-	-	30	-	-	7,1 E+17
**NF2**	90	308,5	-	137,5	15	-	15	2,7 E+17
**NF3**	45+45	304,9	198,6	105,3	11,4	12,0	12,92	2,6 E+17
**IF2**	120	211,8	-	358,5	15,6	-	16,46	4,4 E+17
**IF3**	120	200,2	260,7	317,1	9,3	9,8	9,84	3,2 E+17
